# Robotic surgery for thymic cysts: clinical features, management, and results of a multicentric study

**DOI:** 10.1007/s13304-024-01895-3

**Published:** 2024-05-30

**Authors:** Giacomo Cusumano, Elisa Meacci, Gaetano Romano, Marco Cavaleri, Maria Teresa Congedo, Federico Davini, Stefano Margaritora, Alberto Terminella, Franca Melfi

**Affiliations:** 1 Division of General Thoracic Surgery, University of Catania-“Policlinico-San Marco” University Hospital, Via Santa Sofia 78, 95100 Catania, Italy; 2 Department of General Thoracic Surgery, Fondazione Policlinico Universitario “A. Gemelli”, IRCCS, Università Cattolica del Sacro Cuore, Rome, Italy; 3 Minimally Invasive and Robotic Thoracic Surgery, University Hospital of Pisa, Pisa, Italy; 4 Department of Anesthesia, “Policlinico-San Marco” University Hospital, Catania, Italy

**Keywords:** Thymic cyst, Robotics, Thymectomy, Thymoma, Mediastinal tumors

## Abstract

Thymic cysts are rare, radiological diagnosis is often incidental, and cysts seldom assume clinical relevance for symptoms of compression. Thymoma were occasionally found inside both complex and simple thymic cysts. Given the challenges in accurately clinical diagnosing and since the occasionally discovering of thymoma inside both complex and simple thymic cysts, the management of thymic cysts remains controversial. Advancements in surgical tools such as robotics, applied to thymic conditions, could potentially transform the approach to thymic cysts. We report one the largest multicentric series of thymic cysts surgically treated with robotic approach, focusing on preoperative findings and surgical results. Cases were gathered from three Italian thoracic surgery centers with homogeneous clinical practice, significant experience in thymic neoplasms, and thoracic robotic skilled. Surgical intervention was indicated for patients with radiological diagnosis of thymic cysts under the following circumstances: the presence of symptoms, concurrent myasthenia gravis, cysts growing in follow-up, and the complexity of the cyst with suspicion of neoplasm. Data were collected and matched according to postoperative and pathological features to identify potential prognostic factors. Population include 57 patients, 29/28 male/female ratio with mean age of 59.46 ± 11.67 years. The average size of the thymic cysts was 29.14 ± 24.53 ranged between 3 and 150 mm. All patients undergone CT scan and mean of values of density was 25.82 ± 11–82 Hounsfield. Surgical procedures were robotic approach in all case including total/extended thymectomy 35 (61.4%) and cyst resection/partial thymectomy 22 (38.6%). There were no mortality or recurrence. Major complications rate was 5.3%. No correlations were observed between preoperative features and complication. Pathological examination revealed microfoci of thymic tumor in four cases. Robot-assisted surgery for thymic cysts showed excellent early clinical outcomes with low rate of postoperative complications also in case of large lesion. Thymic cysts should not be underestimated due to the risk of coexistent thymic neoplasm.

## Introduction

Mediastinal cysts constitute approximately 15–20% of all mediastinal masses, with thymic cysts being a rare subgroup comprising around 5% of these mediastinal cysts [1]. Recently, the utilization of screening programs and coronary artery disease assessment through CT scans has led to an increased identification of incidental mediastinal masses, consequently contributing to the recognition of mediastinal cysts [2].

While the majority of these cysts are asymptomatic and lack clinical significance, there are instances where they can hold importance due to the emergence of symptoms or for the suspicion of mediastinal malignancy. Distinguishing between benign cysts and thymic tumors is often challenging, with cystic thymoma and cystic degeneration thymoma being prevalent. Consequently, thymomas can coexist with both complex and simple cysts, indicating the potential for neoplastic transformation in seemingly benign thymic cysts [3, 4].

Radiological observations frequently lack decisiveness in evaluating mediastinal cystic lesions. As a result, the criterion of growth is unreliable, as certain thymic cysts can exhibit growth during follow-up without harboring malignancy or neoplastic elements. Conversely, growth rates demonstrate significant variability among different histologic subtypes established by the World Health Organization (WHO), with growth rates between thymic cysts and thymic tumors being practically indistinguishable [5].

Surgical resection remains the necessary choice in some patients although the risk of unnecessary thymectomy in a fraction of patients [6]. However, a consensus on the management of thymic cysts remains elusive, and advancements in diagnostic methods and surgical tools such as robotics, applied to mediastinal masses and thymic conditions, could potentially transform the approach to thymic cysts. Robotic surgery provides improved precision and dexterity through articulated instruments, along with three-dimensional visualization. These characteristics have demonstrated their effectiveness, particularly in the treatment of mediastinal pathologies, given the anatomical complexity and limited spaces for maneuvering within the mediastinum. Notably, the popularity of robot-assisted surgery for mediastinal masses has steadily increased, showcasing advantages over both open surgery and traditional thoracoscopy.

We herein report one of the largest multicentric series of thymic cysts surgically treated with robotic approach focusing on indications, preoperative clinical and radiological findings, surgical and pathological outcomes, trying to answer several important and debated questions such as: do thymic cysts need surgical treatment? Why do we need to surgically treat these conditions? Is robotic surgery adequate to treat thymic cysts? Is total thymectomy essential in this disease, or cystectomy and partial thymectomy in the absence of myasthenia gravis is adequate? What are the results of robotic surgery in thymic cysts?

## Materials and methods

The current study is a retrospective multicentric observational study that entails the analysis of pre-existing data gathered from three university hospital centers. The Institutional Review Board of the three Italian hospitals involved in this study approval was preliminarily obtained for research using data derived from standard clinical practice with no experimental interventions applied (IRB code 19/2023/PO). Informed written consent was obtained from each patient to include their information in this study. To collect a consistently homogeneous population to conduct this study, cases were gathered from three Italian thoracic surgery centers with homogeneous clinical practice, significant experience in thymic neoplasms, and thoracic robotic skilled: (i) Minimally Invasive and Robotic Thoracic Surgery, University Hospital of Pisa; (ii) Division of Thoracic Surgery, Catholic University of Rome; and (iii) Thoracic Surgery Unit, University Hospital of Catania. The analysis was focused on 57 patients with radiological diagnosis of thymic cysts treated with robotic surgical approach in the period between March 2014 and April 2022. Patients with history of previous mediastinal tumor were excluded from the study.

Surgical intervention was indicated for patients with radiological diagnosis of thymic cysts under the following circumstances: the presence of symptoms, concurrent myasthenia gravis, cysts growing in follow-up and the complexity of the cyst with suspicion of neoplasm. Population was investigated by reviewing the clinical presentation, histopathological characteristics, and treatment modalities. Surgical data, postoperative results, and pathological issues were also retrospectively collected.

### Surgical technique

**Fig. 1:**
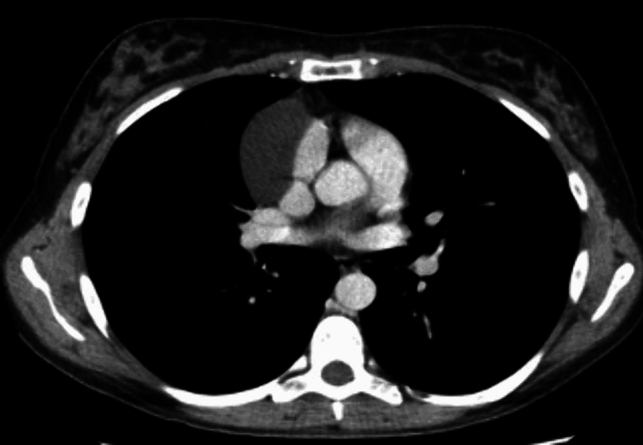
(Thoracic CT scan: thymic cyst)

All procedures were performed using the Xi Da Vinci Robotic System (Intuitive Surgical, Sunnyvale, California, USA) since 2015; before this date, the centers used the Si DaVinci model. The patient, under general anesthesia with selective left intubation, was placed in a supine position the left or right chest elevated by 30°. Routinely, all groups preferred the left-sided approach because it provided excellent exposure of the left phrenic nerve and because of the fact that a larger part of the thymus gland is located on the left side. Right side approach was reserved for patients with lesion predominantly extrinsic on the right side (Fig. 1). Three-port technique was used in the majority of cases and port placement was similar to the port placement for robotic thymectomy. Extended thymectomy was the intervention of choice in patients with suspicion of neoplastic lesion or in case of myasthenia gravis (thymus gland along with all anterior mediastinal and pericardial fat between the two phrenic nerves and from bilateral thyro-thymic ligaments to bilateral pericardiophrenic recesses was resected *en bloc*) [7]. Features considered suspect for thymoma were: multilocular thymic cyst, thick or irregular cyst wall, ill-defined cyst, and severe adhesion with surrounding tissue at chest CT and at intraoperative exploration: Robotic thymectomy are descripted in detail elsewhere [8]. In the other cases, partial thymectomy including cyst removal was performed with the same access and patient’s position. In case lesion of large dimension, the content of the cyst was aspired.

### Statistical analysis

The relationships between categorical variables were evaluated by means of the chi-square test. The Student’s *t*-test and analysis of variance (ANOVA) were applied, respectively, to binary and ordinal predictive variables. The influence of variables which could potentially affect perioperative surgical results (complications, mortality, and duration of intervention, hospitalization and time of drain stay) was tested through univariate logistic regression analysis.

Significant parameters identified by univariate analysis were included in multiple logistic regression (stepwise method; *p* value of 0.05 or less was used for entry into the model and *p* value greater than 0.1 was selected for removal).

All analyses were performed in SPSS version 26.0 (SPSS; Chicago, IL). A *p* value less than 0.05 was considered statistically significant.

## Results

Between March 2014 and April 2022, 57 consecutive patients, 29 (50.9%) males and 28 (49.1%) females, received robotic surgical treatment for thymic cysts at the three institutions reported above. Clinical characteristics of the whole population are reported into Table 1. The mean age and BMI were 59.46 ± 11.67 years and 25.95 ± 3.66, respectively. About 2/3 of patients were asymptomatic for chest pain and dyspnea. Among patients, the first diagnosis of thymic cyst was coronary CT scan done for unspecific chest pain in nine cases (15.8%). Population was affected by comorbidities in 49 cases (86%); myasthenia gravis was present in 15 cases (26.3%) diagnosed by acetylcholine receptors and single fiber electromyography. Eight patients had history of cancer. Finally, seven patients had concomitant cysts at other organs and in particular at kidney. All the thymic cysts were in the anterior mediastinum with their origin into the thymus corpus (*n* = 55) or in the thymic horns (*n* = 5). The average size of the thymic cysts was 29.14 ± 24.53 ranged between 3 and 150 mm. All patients undergone CT scan and mean of values of density was 25.82 ± 11–82 Hounsfield.

**Table 1:** Descriptive statistics

Features	Value and percentage	Mean ± SD
Gender		
Male	29 (50.9%)	
Female	28 (49.1%)	
Age		59.46 ± 11.67 years (range 30–79 years)
BMI		25.95 ± 3.66
Comorbidity	49 (86%)	
Autoimmune diseases	18 (31.6%)	
Myasthenia gravis	15 (26.3%)	
Site of cyst		
Corpus	52 (91.2%)	
Horn	5 (8.8%)	
Cyst dimension		29.14 ± 24.53 mm (range 3–150 mm)
Cysts in other sites	6 (10.5%)	6 kidney
Symptoms	19 (33.3%)	
Coro CT scan	9 (15.8%)	
Hounsfield (HU)		25.82 ± 11–82 HU (range 5–65 HU)
PET scan	13 (22.8%)	
PET positive	4/13 (30.7%)	
MRI	4 (7%)	

Operative and postoperative details are provided in Table 2. In all cases, the cysts were completely removed using the robotic approach, with no instances requiring conversion to thoracotomy or sternotomy, and no intraoperative complications were observed. Extended thymectomy was performed in 35 cases (61%); while in the remaining cases, cyst removal with surrounding tissue (partial thymectomy) was performed.

**Table 2:** Surgical and pathological features

Features	Value and percentage	Mean ± SD
Type of surgery		
Total thymectomy	35 (61.4%)	
Cyst removal	22 (38.6%)	
Side of intervention		
Right	20 (35.1%)	
Left	37 (64.9%)	
Duration of intervention		111.53 ± 50.34 min (range 35–300)
Conversion to thoracotomy or sternotomy	0 (0%)	
Duration of drain stay		2.07 ± 2.55 days (range 1–15)
Hospitalization		3.81 ± 2.85 days (range 2–15)
Major complications	3 (5.3%)	
Minor complications	5 (8.8%)	
Postoperative mortality	0 (0%)	
Type of Cyst		
Simple	35 (61.4%)	
Complex	4 (7%)	
Broncogenic	4 (7%)	
Other	14 (24.6%)	
Thymic hyperplasia	13 (22.8%)	
Thymoma foci	4 (7%)	
Recurrence	0 (0%)	

The mean operation time (from incision to closure) was 111.53 ± 50.34 min, ranging from 35.0 to 300.0 min. The mean duration of chest tube placement was 2.07 ± 2.55 days, with a range of 1.0–15.0 days, and the average postoperative hospital stay was 3.81 ± 2.85 days, ranging from 2.0 to 15.0 days. There were no postoperative mortalities observed.

However, we did encounter and address three significant postoperative complications (5.3%): one case required reoperation for thoracic duct closure due to chylothorax, another case of postoperative anemia was managed conservatively through blood transfusion, and a third case of postoperative respiratory failure was effectively treated with non-invasive ventilation. Additionally, there were minor postoperative complications in 8.8% of cases (*n* = 5), including two cases of fever, one case of urinary tract infection, one case of minor mediastinal hematoma, and one case of persistent hypokalemia.

Pathological analysis revealed that 61% of the cysts were classified as simple, 7% as complex, 7% as bronchogenic intra-thymic, and 25% as other types (mesothelial cyst-pericardial or pleural). Microscopic foci of thymoma were detected in four cases (7%). Additionally, approximately 23% of cases (*n* = 13) showed thymic hyperplasia.

No associations were observed between preoperative features when matched with major and minor complications. Tables 2 and 3 provide detailed information. Complex cysts were significantly associated with the presence of microfoci of thymoma (Pearson χ^2^ = 12.7; *p* = 0.001), as well as a density value at CT scan of more than 40 Hounsfield units (Pearson χ^2^ = 9.23; *p* = 0.001).

**Table 3:** Clinical and perioperative correlation with major and minor complications

Features	Major complication rate (3/57)	Pearson χ^2^	*p* Value
Male gender	3/29	3.057	0.08
Age > 65 years	0/21	1.847	0.17
Comorbidities	3/49	0.517	0.63
Autoimmunity	2/18	1.804	0.18
Myasthenia gravis	2/15	2.659	0.10
Site of cysts (thymus corps)	3/52	0.304	0.58
Type of cysts (simple)	2/35	0.558	0.90
Size of cysts > 3 cm	2/20	1.386	0.23
Symptoms	2/19	1.583	0.21
Type of surgery (total thymectomy)	2/35	0.128	0.93

Features	Minor complication rate (5/57)	Pearson χ^2^	*p* Value
Male gender	3/29	0.183	0.66
Age > 65 years	3/21	1.263	0.26
Comorbidities	4/49	0.162	0.54
Autoimmunity	1/18	0.340	0.56
Myasthenia gravis	1/15	0.113	0.73
Site of cysts (thymus corps)	5/52	0.527	0.46
Type of cysts (simple)	3/35	3.981	0.26
Size of cysts > 3 cm	3/20	1.498	0.22
Symptoms	2/19	0.110	0.74
Type of surgery (total thymectomy)	4/35	0.857	0.65

Hyperplasia was, as expected, associated with autoimmune diseases (Pearson χ^2^ = 6.99; *p* = 0.008) and myasthenia gravis (Pearson χ^2^ = 6.58; *p* = 0.010). However, no association was found with any features related to cysts.

The duration of the surgical procedure was found to be longer for extended thymectomy compared to partial thymus resection (128.46 ± 51.69 min versus 84.59 ± 34.53 min, *p* < 0.001). However, no significant differences were observed between the type of surgery and the occurrence of complications or hospitalization. The duration of drainage was longer in patients who underwent thymectomy compared to those who underwent partial thymus resection (2.64 ± 3.89 days versus 1.71 ± 1.01 days, *p* = 0.09), although this observation only showed a trend toward statistical significance (Table 4). Notably, there were no instances of cyst or thymoma recurrence in any of the cases.

**Table 4:** Unpaired independent *T* test per extended thymectomy vs x cyst resection/partial thymectomy in terms of hospitalization, length of chest drains indwelling, and operation time

Feature	Mean	SD	SE	*p* Value
Duration of intervention				
Cyst resection/partial thymus	**84.59**	**34.536**	**7.363**	** < 0.001**
Extended thymectomy	**128.46**	**51.698**	**8.739**	
Hospital stay				
Cyst resection/partial thymus	3.77	3.545	0.756	0.943
Total thymectomy	3.83	2.320	0.392	
Duration of drain stay				
Total thymectomy	2.64	3.898	0.831	0.09
Cyst resection/partial thymus	1.71	1.017	0.172	

Bold values denote statistical significance at the *p* < 0.05

## Discussion

Thymic cysts are benign mediastinal diseases and constituting approximately 1% of all masses of the anterior mediastinum [9]. In about 2/3 of patients, cysts do not present any symptoms.

They are often incidentally discovered during chest X-rays or chest CT scans conducted for other clinical purposes or during screening programs.

When patients do experience symptoms, the most common complaints include cough, dyspnea, and chest pain. Our data align with the findings in the literature, confirming this pattern with 66.7% of patients being asymptomatic. Notably, about a quarter of these asymptomatic patients incidentally discovered their thymic cysts during coronary CT scans, highlighting the high likelihood of accidental diagnoses linked to screening programs and the increased probability of their discovery in the next future. Other than for the patients with symptoms, surgical resection was indicated for patients with myasthenia gravis, those with cysts exhibiting growth during follow-up, and individuals in whom the cyst displayed suspicious neoplastic features.

No substantial differences were found in the prevalence of cysts based on age and gender. A significant portion of patients had other underlying medical conditions, and approximately 12% of patients had concomitant cysts in other locations, particularly in the kidneys. As observed in other studies, a considerable number of patients had coexisting autoimmune diseases, with roughly a quarter of them having myasthenia gravis. However, no significant associations were identified with respect to thymic hyperplasia, cyst type, or size [10], although hyperplasia was commonly linked to myasthenia gravis and autoimmunity, which was expected. In fact, other studies suggest that complex acquired thymic cysts are more often associated with autoimmune diseases, including myasthenia gravis and thymic hyperplasia [11]. It is worth noting that the data in this field are contradictory, and this topic is far from being clear.

Simple cysts are often congenital and originate from the fetal thymo-pharyngeal tract. They are typically unilocular, with thin walls lined by squamous or small columnar cells, showing no signs of inflammation and containing clear fluid. In contrast, complex cysts are acquired and result from an inflammatory process. They are frequently multiloculated, characterized by thick fibrous walls and the presence of an inflammatory process. Complex cysts may contain turbid fluid or gelatinous material.

While the occurrence of cystic thymomas and cystic degeneration within larger thymomas is common, the discovery of thymomas within the context of cysts is quite exceptional, especially in congenital cysts that originate from the cystic wall. This suggests that neoplastic changes can occur within a simple thymic cyst [3, 4, 9].

The incidence of accidentally discovering thymomas inside cysts is generally considered rare. However, in our study, the rate of thymomas was approximately 7% (4/57), with half of them being found within complex cysts (2/4). Notably, two cases of thymoma also occurred within simple cysts, accounting for about 5% (2/35) of such cases. Other studies have reported similar patterns. For instance, Graber and colleagues reported an incidence of about 5% (2/39) of thymoma foci in patients with simple thymic cysts [9]. Furthermore, Nakamura and colleagues have demonstrated that multilocular/complex thymic cysts associated with thymomas are not as rare as previously believed and may be influenced by inflammatory processes [12].

Thymic lesions may enlarge over time and still be benign, while simple stable congenital cysts can contain neoplasms. Therefore, distinguishing between cystic and solid lesions and classifying cysts is still challenging, both with CT scans and with MRI. The malignant potential of lesions that appear as simple thymic cysts is still undetermined and matter of study [6].

In our study, all patients underwent CT scans, while MRI and 18F-FDG PET/CT (PET) scans were prescribed in selected cases. However, these additional scans did not lead to changes in treatment or strategy for any of the cases, and PET scans did not effectively associate with thymoma or hyperplasia. As suggested by other authors, chest CT scans should be considered the primary diagnostic choice for mediastinal cystic lesions, and MRI or PET–CT may only be prescribed when additional diagnostic information is required [13, 14].

Traditionally, it has been widely accepted that surgical treatment of thymic cysts is indicated for symptomatic cases or those with progressive growth, as obtaining an accurate preoperative diagnosis is often challenging. However, our study reveals that the discovery of thymic neoplasms within cysts is not associated with the presence of symptoms or the size of the lesion. Consequently, it may be necessary to reconsider the indications for surgery in such cases.

Robotic surgery, when applied to mediastinal and thymic diseases, has demonstrated its value by yielding excellent results in both the short and long term. It has proven to be superior to conventional sternotomy in many cases and not inferior to the thoracoscopic approach [15]. The application of robotic surgery in the treatment of thymic cysts has been reported in some case reports or as part of series that include various types of mediastinal masses. However, specific postoperative data regarding the removal of thymic cysts in a large series of patients were primarily focused on those who underwent open surgery (full sternotomy or clamshell sternotomy) rather than minimally invasive surgery [16].

Our study represents the largest series ever published concerning robotic surgery for thymic cysts. The postoperative results are encouraging, with no observed mortality. To the best of our knowledge, the complication rate was lower than in other large experiences reported in the literature prior to our study [17–19]. Nevertheless, the potential superiority of robotic surgery in the management of thymic cysts remains speculative. Robotic surgery, characterized by enhanced precision, dexterity, and three-dimensional visualization, holds significant promise in addressing the complexities associated with mediastinal procedures and has gained widespread acceptance in the treatment of thymic diseases, including thymic cyst management.

However, the scarcity of data on thymic cysts poses a challenge in conducting a robust comparison. The only study, focusing on the VATS approach with a cohort of over a hundred patients [14], encompasses three distinct types of surgical procedures. Furthermore, the postoperative outcomes reported a complication rate of 7 (6.5%), an operation time of 105.0 min (range, 40.0–250.0 min), a mean chest tube indwelling period of 2.3 days (range, 1.0–19.0 days), and a postoperative hospitalization duration of 3.6 days (range, 2.0–21.0 days).

Our data reveal the non-inferiority of the robotic technique in terms of postoperative results.

In our study, we observed a major complication rate of only 5.3%, confirming the safety of the robotic approach for mediastinal cysts. When analyzing the surgical features in our study and comparing them to data from the study with the largest population treated with a thoracoscopic approach of Whang et al. [14], we found that the rates of hospitalization, length of chest drain indwelling, and operation time are comparable. Similar data were found regarding the operation time, which was longer in patients who underwent extended thymectomy compared to cyst resection or partial thymus resection.

However, in our study, no differences were found in terms of complications, hospitalization, and length of chest drain stay when stratifying patients according to cyst resection or partial thymectomy versus extended thymectomy. It is worth noting that these findings may be influenced by the relatively small sample size, and no definitive conclusions can be drawn from this data.

Finally, since all surgical procedures must prioritize safety and efficacy while avoiding additional surgical injury or complications, certain aspects related to the extent of resection need to be considered. First, extended thymectomy is indicated in cases where cysts are associated with myasthenia gravis as a curative treatment.

Thymus-conserving surgery appears to yield similar results as radical thymectomy for early thymomas, even in the case of video-assisted or robotic procedures. Given the incidence rate and the local extent of neoplastic disease, particularly thymoma microfoci, this procedure may be suitable for this group of patients. Certainly, thymic cyst resection or partial thymectomy should be considered for cases of simple thymic cysts due to their low probability of malignancy. In all cases, however, it is essential to ensure the complete resection of thymic lesions.

## Limitations and strengths

Our study is subject to some inherent limitations that are a consequence of the rare nature of the disease. Specifically, the retrospective design of the study, the relatively small number of patients gathered from three different centers, and the lack of long-term follow-up represent the primary weaknesses. Additionally, certain results should be approached with caution due to the limited sample size, particularly in the context of multivariable analysis. On the other hand, this study comprises the largest patient cohort reported in the literature concerning this particular “uncommon disease” treated using a consistent robotic approach. Finally, the significant incidence of malignancy within this series is noteworthy, even in cases of simple thymic cysts. This aspect may, therefore, stimulate a new debate among thoracic surgeons and oncologists. Finally, our study does not encompass a cost-effective analysis. The costs associated with robotic surgery are undeniably higher than those of the thoracoscopic approach. However, contrary to the initial perception that robotic surgery incurs higher costs, it is crucial to underscore the broader economic impact. In the context of rare diseases such as thymic cysts, where specialized expertise and precision are paramount, the advantages inherent in robotic surgery may potentially lead to long-term cost-effectiveness. The diminished requirement for extensive postoperative care, reduced hospitalization durations, and potentially expedited recovery could contribute to an overall reduction in healthcare costs. We anticipate that these costs will decrease in the near future, aligning with the increased adoption of robotic technology.

In conclusion, our study underscores that robot-assisted surgery offers the opportunity to treat patients with thymic cysts, showing excellent postoperative outcomes with a low rate of postoperative complications, even in cases of large cystic lesions. Further research is necessary to assess long-term clinical and oncological outcomes, with a particular focus on extended follow-up.

## Data Availability

Access to the data is possible but subject to approval from the three involved centers and their individual decisions.
